# Bis[(2-pyrid­yl)(2-pyridyl­amino)­methano­lato]manganese(III) nitrate

**DOI:** 10.1107/S1600536811014371

**Published:** 2011-04-22

**Authors:** Shuai Ding, Congjun Xia, Yufei Ji, Zhiliang Liu, Yong Ding

**Affiliations:** a College of Chemistry and Chemical Engineering, Inner Mongolia University, Hohhot 010021, People’s Republic of China; b Yanzhou Coal Mining Logistics Company Ltd, People’s Republic of China

## Abstract

The Mn^III^ atom in the title complex, [Mn(C_11_H_10_N_3_O)_2_]NO_3_, is coordinated by the two tridentate (2-pyrid­yl)(2-pyridyl­amino)­methano­late ligands, forming a six-coordinate environment. The four pyridyl N atoms constitute the equatorial plane on which the manganese(III) ion lies; the coordination plane suffers a slight distortion as indicated by the average plane deviation of 0.058 Å. The methano­late O atoms occupy the axial positions. The coordination geometry is thus octa­hedral. In the title compound, the cations are linked by nitrate anions *via* N—H⋯O hydrogen bonds to form one-dimensional chains. Moreover, the one-dimensional structure is stabilized by inter­molecular edge-to-face aromatic π–π inter­actions with a center-of-inversion at a distance of *ca* 4.634 Å.

## Related literature

For related structures, see: Adams *et al.* (2005 ▶); Liu *et al.* (2008 ▶); Arulsamy & Hongson (1994 ▶).
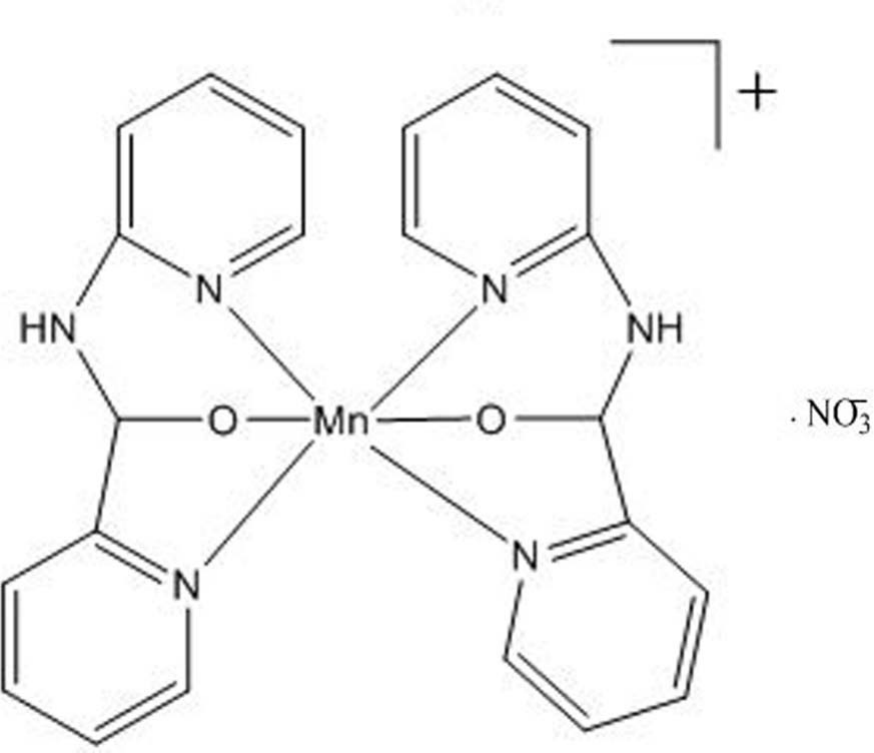


## Experimental

### 

#### Crystal data

[Mn(C_11_H_10_N_3_O)_2_]NO_3_
*M* *_r_* = 517.39
Monoclinic, 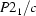

*a* = 12.889 (3) Å
*b* = 10.931 (2) Å
*c* = 19.309 (6) Å
β = 123.34 (2)°
*V* = 2272.7 (10) Å^3^
*Z* = 4
Mo *K*α radiation
μ = 0.63 mm^−1^
*T* = 153 K
0.10 × 0.05 × 0.05 mm

#### Data collection

Rigaku Saturn CCD area-detector diffractometer
Absorption correction: multi-scan (*CrystalClear*; Rigaku/MSC, 2005 ▶) *T*_min_ = 0.963, *T*_max_ = 0.969
16544 measured reflections
5743 independent reflections
4648 reflections with *I* > 2σ(*I*)
*R*_int_ = 0.021

#### Refinement

*R*[*F* ^2^ > 2σ(*F* ^2^)] = 0.051
*wR*(*F* ^2^) = 0.166
*S* = 1.07
5743 reflections
316 parameters
H-atom parameters constrained
Δρ_max_ = 1.12 e Å^−3^
Δρ_min_ = −0.47 e Å^−3^

### 

Data collection: *CrystalClear* (Rigaku/MSC, 2005 ▶); cell refinement: *CrystalClear*; data reduction: *CrystalClear*; program(s) used to solve structure: *SHELXS97* (Sheldrick, 2008 ▶); program(s) used to refine structure: *SHELXL97* (Sheldrick, 2008 ▶); molecular graphics: *DIAMOND* (Brandenburg & Putz, 2006 ▶) and *XP* (Siemens, 1994 ▶); software used to prepare material for publication: *publCIF* (Westrip, 2010 ▶).

## Supplementary Material

Crystal structure: contains datablocks I, global. DOI: 10.1107/S1600536811014371/zk2005sup1.cif

Structure factors: contains datablocks I. DOI: 10.1107/S1600536811014371/zk2005Isup2.hkl

Additional supplementary materials:  crystallographic information; 3D view; checkCIF report

## Figures and Tables

**Table 1:** Selected bond lengths (Å)

Mn1—O2	1.8428 (17)
Mn1—O1	1.8488 (17)
Mn1—N6	2.111 (2)
Mn1—N4	2.139 (2)
Mn1—N3	2.179 (2)
Mn1—N5	2.202 (2)

**Table 2:** Hydrogen-bond geometry (Å, °)

*D*—H⋯*A*	*D*—H	H⋯*A*	*D*⋯*A*	*D*—H⋯*A*
N1—H1⋯O3^i^	0.86	2.08	2.931 (4)	171
N2—H2⋯O4^ii^	0.86	2.07	2.902 (4)	164

Symmetry codes: (i) 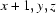; (ii) 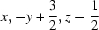.

**Fig. 1.**
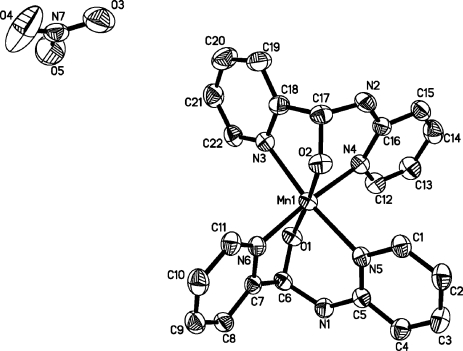
The molecular structure of the title compound, with atom labels and 30% probability displacement ellipsoids.

**Fig. 2.**
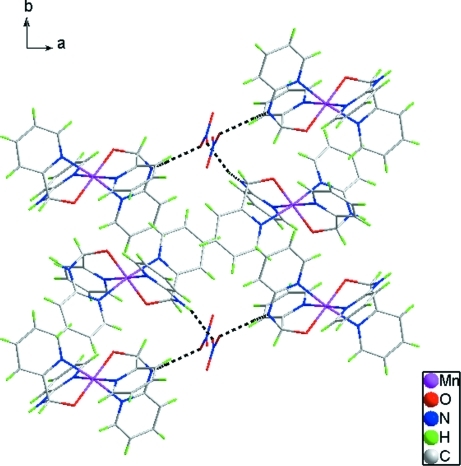
Part of the crystal structure of the title compound showing hydrogen bond as dashed lines.

**Fig. 3.**
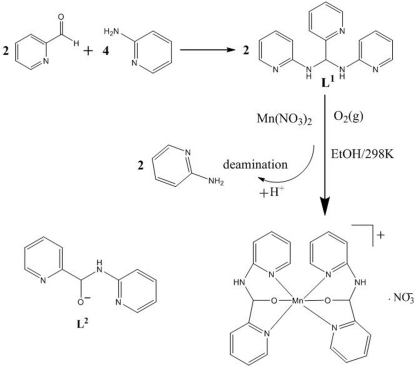
The formation of the title compound.

## supplementary crystallographic information

### Comment

Selected bond distances and angles are given in Table 1. (2-pyridyl)(2-pyridylamino)-methanolato is a transmutative ligand. There is only four complexes with this ligand reported, such as those of Mn^III^ (Liu *et al.* 2008), Co^III^ (Adams *et al.* 2005) and Mn^III^, Fe^III^ (Arulsamy & Hongson 1994). We report herein the synthesis and structure of the title compound. The crystal structure of the complex cation shows two tridentate (2-pyridyl)bis(2-pyridylamino)-methanolate ligands coordinated facially to the Mn^III^ ion to form a distorted octahedral geometry. The Mn^III^ resides on a pseudo-twofold axis of symmetry and on an equatorial plane formed by the pyridyl N atoms of the two ligands (Fig. 2, Table 2).

### Experimental

*L*^1^ was synthesized following literature procedures (Arulsamy & Hongson 1994). 50% Mn(NO_3_)_2_ (0.356 g, 1 mmol) was added to a solution of *L*^1^ (0.277 g, 1 mmol) in ehanol (20 ml); a brown solution formed over time with continuous stirring at room temperature. This solution was left to evaporate slowly at room temperature. After one week, brown block crystals of the title compound were isolated (yield: 0.1662 g, 60%). Microanalysis found: C 50.91, H 3.89, N 18.90%; calculated for C_22_H_20_MnN_7_O_5_ (Mr = 517.39): C 51.02, H 3.86, N 18.94%.

### Refinement

The H atoms were placed in geometrically idealized positions (C—H = 0.95 Å and O—H = 0.82–0.84 Å), with *U*_iso_(H) = 1.2Ueq(C) and *U*_iso_(H) = 1.5Ueq(O).

### Crystal data

**Table:**

[Mn(C_11_H_10_N_3_O)_2_]NO_3_	*F*(000) = 1064
*M**_r_* = 517.39	*D*_x_ = 1.512 Mg m^−^^3^
Monoclinic, *P*2_1_/*c*	Mo *K*α radiation, λ = 0.71073 Å
*a* = 12.889 (3) Å	Cell parameters from 3732 reflections
*b* = 10.931 (2) Å	θ = 1.9–27.9°
*c* = 19.309 (6) Å	µ = 0.63 mm^−^^1^
β = 123.34 (2)°	*T* = 153 K
*V* = 2272.7 (10) Å^3^	Block, brown
*Z* = 4	0.1 × 0.05 × 0.05 mm

### Data collection

**Table:**

Rigaku Saturn CCD area-detector diffractometer	5743 independent reflections
Radiation source: fine-focus sealed tube	4648 reflections with *I* > 2σ(*I*)
graphite	*R*_int_ = 0.021
Detector resolution: 7.31 pixels mm^-1^	θ_max_ = 28.5°, θ_min_ = 1.9°
ω and φ scans	*h* = −13→17
Absorption correction: multi-scan (*CrystalClear*; Rigaku/MSC, 2005)	*k* = −13→14
*T*_min_ = 0.963, *T*_max_ = 0.969	*l* = −25→25
16544 measured reflections	

### Refinement

**Table:**

Refinement on *F*^2^	Primary atom site location: structure-invariant direct methods
Least-squares matrix: full	Secondary atom site location: difference Fourier map
*R*[*F*^2^ > 2σ(*F*^2^)] = 0.051	Hydrogen site location: inferred from neighbouring sites
*wR*(*F*^2^) = 0.166	H-atom parameters constrained
*S* = 1.07	*w* = 1/[σ^2^(*F*_o_^2^) + (0.091*P*)^2^ + 1.2493*P*] where *P* = (*F*_o_^2^ + 2*F*_c_^2^)/3
5743 reflections	(Δ/σ)_max_ = 0.001
316 parameters	Δρ_max_ = 1.12 e Å^−^^3^
0 restraints	Δρ_min_ = −0.47 e Å^−^^3^

### Special details

**Table:**

Geometry. All e.s.d.'s (except the e.s.d. in the dihedral angle between two l.s. planes) are estimated using the full covariance matrix. The cell e.s.d.'s are taken into account individually in the estimation of e.s.d.'s in distances, angles and torsion angles; correlations between e.s.d.'s in cell parameters are only used when they are defined by crystal symmetry. An approximate (isotropic) treatment of cell e.s.d.'s is used for estimating e.s.d.'s involving l.s. planes.
Refinement. Refinement of *F*^2^ against ALL reflections. The weighted *R*-factor *wR* and goodness of fit *S* are based on *F*^2^, conventional *R*-factors *R* are based on *F*, with *F* set to zero for negative *F*^2^. The threshold expression of *F*^2^ > σ(*F*^2^) is used only for calculating *R*-factors(gt) *etc*. and is not relevant to the choice of reflections for refinement. *R*-factors based on *F*^2^ are statistically about twice as large as those based on *F*, and *R*- factors based on ALL data will be even larger.

### Fractional atomic coordinates and isotropic or equivalent isotropic displacement parameters (Å^2^)

**Table:**

	*x*	*y*	*z*	*U*_iso_*/*U*_eq_	
Mn1	0.91585 (3)	0.67947 (3)	0.65817 (2)	0.04290 (14)	
O1	1.01651 (17)	0.54585 (15)	0.67793 (11)	0.0507 (4)	
O2	0.81804 (17)	0.81050 (16)	0.64742 (13)	0.0530 (4)	
N1	1.2075 (2)	0.6381 (2)	0.72255 (14)	0.0541 (5)	
H1	1.2780	0.6098	0.7351	0.065*	
N2	0.6572 (2)	0.7950 (2)	0.50627 (16)	0.0608 (6)	
H2	0.5951	0.8410	0.4732	0.073*	
N3	0.7563 (2)	0.5819 (2)	0.64167 (13)	0.0501 (5)	
N4	0.8165 (2)	0.66973 (18)	0.52595 (14)	0.0485 (5)	
N5	1.0588 (2)	0.79115 (19)	0.65910 (12)	0.0459 (4)	
N6	1.0294 (2)	0.68841 (18)	0.78880 (13)	0.0461 (4)	
C1	1.0262 (3)	0.9030 (2)	0.62310 (17)	0.0554 (6)	
H1A	0.9486	0.9339	0.6065	0.066*	
C2	1.1011 (3)	0.9718 (3)	0.6102 (2)	0.0674 (8)	
H2A	1.0760	1.0486	0.5857	0.081*	
C3	1.2157 (3)	0.9253 (3)	0.6342 (2)	0.0710 (8)	
H3	1.2687	0.9709	0.6257	0.085*	
C4	1.2511 (3)	0.8141 (3)	0.6699 (2)	0.0597 (7)	
H4	1.3276	0.7816	0.6852	0.072*	
C5	1.1708 (2)	0.7481 (2)	0.68361 (15)	0.0474 (5)	
C6	1.1368 (2)	0.5659 (2)	0.74488 (15)	0.0479 (5)	
H6	1.1777	0.4864	0.7648	0.058*	
C7	1.1348 (2)	0.6272 (2)	0.81495 (15)	0.0453 (5)	
C8	1.2290 (3)	0.6237 (3)	0.89677 (17)	0.0557 (6)	
H8	1.3017	0.5810	0.9138	0.067*	
C9	1.2149 (3)	0.6842 (3)	0.95381 (19)	0.0637 (7)	
H9	1.2775	0.6820	1.0099	0.076*	
C10	1.1077 (3)	0.7475 (3)	0.92669 (19)	0.0670 (8)	
H10	1.0966	0.7894	0.9641	0.080*	
C11	1.0163 (3)	0.7485 (3)	0.84341 (18)	0.0571 (6)	
H11	0.9437	0.7923	0.8249	0.068*	
C12	0.8687 (3)	0.6069 (3)	0.49241 (18)	0.0605 (7)	
H12	0.9378	0.5586	0.5273	0.073*	
C13	0.8261 (3)	0.6101 (3)	0.4106 (2)	0.0727 (9)	
H13	0.8643	0.5650	0.3898	0.087*	
C14	0.7234 (4)	0.6832 (3)	0.3591 (2)	0.0752 (9)	
H14	0.6922	0.6884	0.3028	0.090*	
C15	0.6696 (3)	0.7461 (3)	0.39065 (18)	0.0637 (7)	
H15	0.6019	0.7963	0.3565	0.076*	
C16	0.7153 (2)	0.7364 (2)	0.47519 (16)	0.0507 (6)	
C17	0.6923 (2)	0.7859 (3)	0.59136 (18)	0.0541 (6)	
H17	0.6442	0.8464	0.5999	0.065*	
C18	0.6618 (3)	0.6604 (3)	0.60818 (18)	0.0539 (6)	
C19	0.5459 (3)	0.6273 (4)	0.5890 (2)	0.0768 (9)	
H19	0.4810	0.6834	0.5665	0.092*	
C20	0.5287 (4)	0.5062 (4)	0.6047 (3)	0.0914 (12)	
H20	0.4521	0.4810	0.5935	0.110*	
C21	0.6239 (4)	0.4266 (4)	0.6359 (2)	0.0792 (10)	
H21	0.6129	0.3454	0.6449	0.095*	
C22	0.7368 (3)	0.4663 (3)	0.65425 (18)	0.0625 (7)	
H22	0.8023	0.4110	0.6763	0.075*	
O3	0.4514 (3)	0.5305 (4)	0.7831 (3)	0.1256 (13)	
O4	0.4849 (5)	0.5147 (5)	0.9012 (3)	0.193 (3)	
O5	0.5144 (4)	0.3663 (4)	0.8475 (3)	0.1308 (13)	
N7	0.4830 (2)	0.4735 (3)	0.84692 (19)	0.0689 (7)	

### Atomic displacement parameters (Å^2^)

**Table:**

	*U*^11^	*U*^22^	*U*^33^	*U*^12^	*U*^13^	*U*^23^
Mn1	0.0444 (2)	0.0363 (2)	0.0444 (2)	0.00432 (13)	0.02212 (17)	−0.00227 (13)
O1	0.0534 (9)	0.0371 (8)	0.0504 (9)	0.0062 (7)	0.0214 (8)	−0.0026 (7)
O2	0.0506 (9)	0.0411 (9)	0.0653 (11)	0.0031 (7)	0.0306 (9)	−0.0091 (8)
N1	0.0540 (12)	0.0532 (12)	0.0613 (13)	0.0145 (10)	0.0357 (11)	0.0103 (10)
N2	0.0547 (12)	0.0543 (13)	0.0662 (14)	0.0160 (10)	0.0287 (11)	0.0123 (11)
N3	0.0530 (11)	0.0481 (11)	0.0484 (11)	0.0031 (9)	0.0274 (9)	−0.0006 (9)
N4	0.0534 (11)	0.0406 (10)	0.0499 (11)	0.0042 (8)	0.0275 (10)	−0.0014 (8)
N5	0.0535 (11)	0.0407 (10)	0.0449 (10)	0.0065 (8)	0.0278 (9)	0.0018 (8)
N6	0.0521 (11)	0.0401 (10)	0.0481 (10)	0.0009 (8)	0.0288 (9)	0.0025 (8)
C1	0.0655 (16)	0.0454 (13)	0.0576 (14)	0.0096 (12)	0.0353 (13)	0.0061 (11)
C2	0.089 (2)	0.0502 (15)	0.0748 (19)	0.0079 (15)	0.0525 (18)	0.0130 (14)
C3	0.089 (2)	0.0633 (18)	0.084 (2)	−0.0020 (16)	0.062 (2)	0.0081 (16)
C4	0.0639 (16)	0.0626 (17)	0.0637 (17)	0.0020 (13)	0.0420 (14)	−0.0004 (13)
C5	0.0548 (13)	0.0470 (13)	0.0433 (12)	0.0044 (10)	0.0288 (10)	−0.0025 (9)
C6	0.0503 (12)	0.0384 (11)	0.0491 (12)	0.0090 (10)	0.0234 (10)	0.0040 (9)
C7	0.0508 (12)	0.0366 (11)	0.0483 (12)	−0.0019 (9)	0.0271 (10)	0.0040 (9)
C8	0.0529 (14)	0.0542 (15)	0.0511 (14)	−0.0022 (12)	0.0229 (11)	0.0059 (11)
C9	0.0701 (18)	0.0674 (18)	0.0454 (14)	−0.0106 (14)	0.0266 (13)	−0.0023 (12)
C10	0.087 (2)	0.0666 (19)	0.0558 (16)	−0.0055 (16)	0.0444 (16)	−0.0067 (14)
C11	0.0685 (16)	0.0524 (15)	0.0614 (16)	0.0036 (12)	0.0427 (14)	0.0024 (12)
C12	0.0697 (17)	0.0561 (16)	0.0582 (15)	0.0077 (13)	0.0368 (14)	−0.0045 (12)
C13	0.086 (2)	0.075 (2)	0.0596 (17)	0.0011 (17)	0.0414 (17)	−0.0122 (15)
C14	0.090 (2)	0.076 (2)	0.0496 (16)	−0.0095 (18)	0.0321 (16)	−0.0040 (14)
C15	0.0597 (16)	0.0601 (17)	0.0523 (15)	−0.0043 (13)	0.0187 (13)	0.0059 (12)
C16	0.0476 (12)	0.0407 (12)	0.0546 (14)	−0.0023 (10)	0.0222 (11)	0.0037 (10)
C17	0.0489 (13)	0.0466 (13)	0.0672 (16)	0.0091 (11)	0.0322 (12)	−0.0013 (12)
C18	0.0505 (13)	0.0545 (14)	0.0566 (14)	0.0037 (11)	0.0293 (12)	−0.0015 (11)
C19	0.0529 (16)	0.081 (2)	0.092 (2)	0.0022 (16)	0.0371 (17)	0.0055 (19)
C20	0.072 (2)	0.091 (3)	0.112 (3)	−0.022 (2)	0.050 (2)	−0.004 (2)
C21	0.086 (2)	0.067 (2)	0.074 (2)	−0.0146 (18)	0.0376 (18)	0.0082 (16)
C22	0.0710 (18)	0.0527 (15)	0.0560 (15)	0.0021 (13)	0.0300 (14)	0.0075 (12)
O3	0.088 (2)	0.123 (3)	0.140 (3)	0.014 (2)	0.046 (2)	0.044 (2)
O4	0.228 (5)	0.236 (5)	0.214 (5)	−0.173 (4)	0.183 (4)	−0.163 (4)
O5	0.141 (3)	0.109 (3)	0.160 (4)	0.031 (2)	0.094 (3)	0.033 (3)
N7	0.0434 (12)	0.0718 (18)	0.0776 (18)	−0.0087 (12)	0.0245 (12)	0.0023 (14)

### Geometric parameters (Å, °)

**Table:**

Mn1—O2	1.8428 (17)	C6—H6	0.9800
Mn1—O1	1.8488 (17)	C7—C8	1.365 (4)
Mn1—N6	2.111 (2)	C8—C9	1.380 (4)
Mn1—N4	2.139 (2)	C8—H8	0.9300
Mn1—N3	2.179 (2)	C9—C10	1.366 (5)
Mn1—N5	2.202 (2)	C9—H9	0.9300
O1—C6	1.385 (3)	C10—C11	1.375 (4)
O2—C17	1.393 (3)	C10—H10	0.9300
N1—C5	1.358 (3)	C11—H11	0.9300
N1—C6	1.439 (3)	C12—C13	1.358 (4)
N1—H1	0.8600	C12—H12	0.9300
N2—C16	1.352 (4)	C13—C14	1.390 (5)
N2—C17	1.448 (4)	C13—H13	0.9300
N2—H2	0.8601	C14—C15	1.338 (5)
N3—C18	1.332 (3)	C14—H14	0.9300
N3—C22	1.336 (4)	C15—C16	1.402 (4)
N4—C16	1.337 (3)	C15—H15	0.9300
N4—C12	1.351 (3)	C17—C18	1.511 (4)
N5—C5	1.333 (3)	C17—H17	0.9800
N5—C1	1.354 (3)	C18—C19	1.376 (4)
N6—C11	1.329 (3)	C19—C20	1.401 (6)
N6—C7	1.339 (3)	C19—H19	0.9300
C1—C2	1.350 (4)	C20—C21	1.346 (6)
C1—H1A	0.9300	C20—H20	0.9300
C2—C3	1.380 (5)	C21—C22	1.365 (5)
C2—H2A	0.9300	C21—H21	0.9300
C3—C4	1.348 (4)	C22—H22	0.9300
C3—H3	0.9300	O3—N7	1.233 (4)
C4—C5	1.401 (4)	O4—N7	1.129 (4)
C4—H4	0.9300	O5—N7	1.237 (5)
C6—C7	1.523 (3)		
			
O2—Mn1—O1	175.06 (9)	C7—C6—H6	108.1
O2—Mn1—N6	94.63 (8)	N6—C7—C8	121.6 (2)
O1—Mn1—N6	80.96 (8)	N6—C7—C6	113.2 (2)
O2—Mn1—N4	88.88 (9)	C8—C7—C6	125.2 (2)
O1—Mn1—N4	95.67 (8)	C7—C8—C9	119.1 (3)
N6—Mn1—N4	174.62 (8)	C7—C8—H8	120.5
O2—Mn1—N3	80.32 (8)	C9—C8—H8	120.5
O1—Mn1—N3	98.18 (8)	C10—C9—C8	119.1 (3)
N6—Mn1—N3	100.15 (8)	C10—C9—H9	120.5
N4—Mn1—N3	84.45 (9)	C8—C9—H9	120.5
O2—Mn1—N5	95.03 (8)	C9—C10—C11	119.2 (3)
O1—Mn1—N5	86.97 (8)	C9—C10—H10	120.4
N6—Mn1—N5	86.39 (8)	C11—C10—H10	120.4
N4—Mn1—N5	89.24 (8)	N6—C11—C10	121.5 (3)
N3—Mn1—N5	172.21 (8)	N6—C11—H11	119.2
C6—O1—Mn1	111.44 (14)	C10—C11—H11	119.2
C17—O2—Mn1	111.68 (15)	N4—C12—C13	123.7 (3)
C5—N1—C6	124.5 (2)	N4—C12—H12	118.2
C5—N1—H1	117.9	C13—C12—H12	118.2
C6—N1—H1	117.6	C12—C13—C14	117.7 (3)
C16—N2—C17	124.6 (2)	C12—C13—H13	121.1
C16—N2—H2	117.6	C14—C13—H13	121.1
C17—N2—H2	117.8	C15—C14—C13	119.8 (3)
C18—N3—C22	119.0 (3)	C15—C14—H14	120.1
C18—N3—Mn1	107.03 (18)	C13—C14—H14	120.1
C22—N3—Mn1	133.9 (2)	C14—C15—C16	120.0 (3)
C16—N4—C12	117.9 (2)	C14—C15—H15	120.0
C16—N4—Mn1	123.43 (18)	C16—C15—H15	120.0
C12—N4—Mn1	117.92 (19)	N4—C16—N2	119.2 (2)
C5—N5—C1	118.1 (2)	N4—C16—C15	120.8 (3)
C5—N5—Mn1	123.03 (17)	N2—C16—C15	120.0 (3)
C1—N5—Mn1	118.31 (18)	O2—C17—N2	111.9 (2)
C11—N6—C7	119.5 (2)	O2—C17—C18	109.7 (2)
C11—N6—Mn1	131.58 (19)	N2—C17—C18	110.5 (2)
C7—N6—Mn1	108.72 (16)	O2—C17—H17	108.2
C2—C1—N5	123.1 (3)	N2—C17—H17	108.2
C2—C1—H1A	118.5	C18—C17—H17	108.2
N5—C1—H1A	118.5	N3—C18—C19	121.9 (3)
C1—C2—C3	118.4 (3)	N3—C18—C17	114.4 (2)
C1—C2—H2A	120.8	C19—C18—C17	123.6 (3)
C3—C2—H2A	120.8	C18—C19—C20	117.9 (3)
C4—C3—C2	120.2 (3)	C18—C19—H19	121.1
C4—C3—H3	119.9	C20—C19—H19	121.1
C2—C3—H3	119.9	C21—C20—C19	119.6 (3)
C3—C4—C5	118.9 (3)	C21—C20—H20	120.2
C3—C4—H4	120.6	C19—C20—H20	120.2
C5—C4—H4	120.6	C20—C21—C22	119.3 (3)
N5—C5—N1	119.2 (2)	C20—C21—H21	120.3
N5—C5—C4	121.3 (2)	C22—C21—H21	120.3
N1—C5—C4	119.4 (2)	N3—C22—C21	122.2 (3)
O1—C6—N1	112.0 (2)	N3—C22—H22	118.9
O1—C6—C7	110.0 (2)	C21—C22—H22	118.9
N1—C6—C7	110.3 (2)	O4—N7—O3	123.5 (5)
O1—C6—H6	108.1	O4—N7—O5	122.0 (5)
N1—C6—H6	108.1	O3—N7—O5	114.5 (4)
			
O2—Mn1—O1—C6	−62.4 (10)	C1—N5—C5—C4	−2.4 (4)
N6—Mn1—O1—C6	−35.28 (17)	Mn1—N5—C5—C4	168.7 (2)
N4—Mn1—O1—C6	140.48 (17)	C6—N1—C5—N5	−4.3 (4)
N3—Mn1—O1—C6	−134.33 (17)	C6—N1—C5—C4	175.8 (2)
N5—Mn1—O1—C6	51.54 (17)	C3—C4—C5—N5	2.7 (4)
O1—Mn1—O2—C17	−108.0 (10)	C3—C4—C5—N1	−177.4 (3)
N6—Mn1—O2—C17	−134.89 (19)	Mn1—O1—C6—N1	−80.4 (2)
N4—Mn1—O2—C17	49.19 (19)	Mn1—O1—C6—C7	42.7 (2)
N3—Mn1—O2—C17	−35.37 (19)	C5—N1—C6—O1	53.1 (3)
N5—Mn1—O2—C17	138.33 (19)	C5—N1—C6—C7	−69.9 (3)
O2—Mn1—N3—C18	18.63 (18)	C11—N6—C7—C8	0.9 (4)
O1—Mn1—N3—C18	−166.13 (18)	Mn1—N6—C7—C8	176.2 (2)
N6—Mn1—N3—C18	111.65 (18)	C11—N6—C7—C6	−178.6 (2)
N4—Mn1—N3—C18	−71.18 (18)	Mn1—N6—C7—C6	−3.2 (2)
N5—Mn1—N3—C18	−35.1 (6)	O1—C6—C7—N6	−23.8 (3)
O2—Mn1—N3—C22	−165.0 (3)	N1—C6—C7—N6	100.3 (2)
O1—Mn1—N3—C22	10.2 (3)	O1—C6—C7—C8	156.8 (2)
N6—Mn1—N3—C22	−72.0 (3)	N1—C6—C7—C8	−79.1 (3)
N4—Mn1—N3—C22	105.2 (3)	N6—C7—C8—C9	0.3 (4)
N5—Mn1—N3—C22	141.3 (5)	C6—C7—C8—C9	179.6 (3)
O2—Mn1—N4—C16	−7.8 (2)	C7—C8—C9—C10	−0.9 (4)
O1—Mn1—N4—C16	170.3 (2)	C8—C9—C10—C11	0.5 (5)
N6—Mn1—N4—C16	−138.6 (8)	C7—N6—C11—C10	−1.3 (4)
N3—Mn1—N4—C16	72.6 (2)	Mn1—N6—C11—C10	−175.4 (2)
N5—Mn1—N4—C16	−102.8 (2)	C9—C10—C11—N6	0.6 (5)
O2—Mn1—N4—C12	162.2 (2)	C16—N4—C12—C13	1.7 (5)
O1—Mn1—N4—C12	−19.7 (2)	Mn1—N4—C12—C13	−168.8 (3)
N6—Mn1—N4—C12	31.3 (9)	N4—C12—C13—C14	0.5 (5)
N3—Mn1—N4—C12	−117.4 (2)	C12—C13—C14—C15	−0.7 (5)
N5—Mn1—N4—C12	67.1 (2)	C13—C14—C15—C16	−1.2 (5)
O2—Mn1—N5—C5	164.94 (19)	C12—N4—C16—N2	176.5 (3)
O1—Mn1—N5—C5	−10.53 (19)	Mn1—N4—C16—N2	−13.5 (3)
N6—Mn1—N5—C5	70.60 (19)	C12—N4—C16—C15	−3.6 (4)
N4—Mn1—N5—C5	−106.25 (19)	Mn1—N4—C16—C15	166.3 (2)
N3—Mn1—N5—C5	−142.1 (5)	C17—N2—C16—N4	−3.6 (4)
O2—Mn1—N5—C1	−24.0 (2)	C17—N2—C16—C15	176.5 (3)
O1—Mn1—N5—C1	160.53 (19)	C14—C15—C16—N4	3.5 (4)
N6—Mn1—N5—C1	−118.35 (19)	C14—C15—C16—N2	−176.6 (3)
N4—Mn1—N5—C1	64.80 (19)	Mn1—O2—C17—N2	−77.3 (2)
N3—Mn1—N5—C1	28.9 (7)	Mn1—O2—C17—C18	45.6 (3)
O2—Mn1—N6—C11	13.3 (2)	C16—N2—C17—O2	52.2 (4)
O1—Mn1—N6—C11	−164.4 (2)	C16—N2—C17—C18	−70.3 (3)
N4—Mn1—N6—C11	143.9 (8)	C22—N3—C18—C19	2.5 (4)
N3—Mn1—N6—C11	−67.7 (2)	Mn1—N3—C18—C19	179.5 (3)
N5—Mn1—N6—C11	108.1 (2)	C22—N3—C18—C17	−176.0 (2)
O2—Mn1—N6—C7	−161.26 (16)	Mn1—N3—C18—C17	1.0 (3)
O1—Mn1—N6—C7	20.99 (16)	O2—C17—C18—N3	−28.3 (3)
N4—Mn1—N6—C7	−30.6 (9)	N2—C17—C18—N3	95.6 (3)
N3—Mn1—N6—C7	117.77 (16)	O2—C17—C18—C19	153.3 (3)
N5—Mn1—N6—C7	−66.51 (16)	N2—C17—C18—C19	−82.9 (4)
C5—N5—C1—C2	0.8 (4)	N3—C18—C19—C20	−1.0 (5)
Mn1—N5—C1—C2	−170.7 (2)	C17—C18—C19—C20	177.3 (3)
N5—C1—C2—C3	0.6 (5)	C18—C19—C20—C21	−1.3 (6)
C1—C2—C3—C4	−0.2 (5)	C19—C20—C21—C22	2.0 (6)
C2—C3—C4—C5	−1.3 (5)	C18—N3—C22—C21	−1.7 (4)
C1—N5—C5—N1	177.7 (2)	Mn1—N3—C22—C21	−177.7 (2)
Mn1—N5—C5—N1	−11.3 (3)	C20—C21—C22—N3	−0.5 (6)

### Hydrogen-bond geometry (Å, °)

**Table:**

*D*—H···*A*	*D*—H	H···*A*	*D*···*A*	*D*—H···*A*
N1—H1···O3^i^	0.86	2.08	2.931 (4)	171.
N2—H2···O4^ii^	0.86	2.07	2.902 (4)	164.

Symmetry codes: (i) *x*+1, *y*, *z*; (ii) *x*, −*y*+3/2, *z*−1/2.
